# Ultrasound findings in painful spastic hip. Muscle thickness in children with cerebral palsy

**DOI:** 10.1186/s12891-023-06610-8

**Published:** 2023-06-22

**Authors:** Claudia Guízar-Sánchez, Cristina Hernández-Díaz, Diana Guízar-Sánchez, Ana Victoria Meza-Sánchez, Alejandra Torres-Serrano, María Elena Camacho Cruz, Lucio Ventura-Ríos

**Affiliations:** 1 Pediatric Physical Medicine and Rehabilitation Service, Hospital de Pediatría Centro Médico Nacional Siglo XXI (CMNSXXI), Mexico City, México; 2 Rheumatology Department, Hospital Juárez de México, Mexico City, México; 3 Physiology Department, Faculty of Medicine, Universidad Nacional Autónoma de México, Mexico City, México Av. Universidad 3004, Col. Copilco Universidad, Alcaldía Coyoacán, Cd. Universitaria,; 4 Radiology and Imaging Department, Instituto Nacional de Ciencias Médicas y Nutricion Salvador Zubirán., Ciudad de México, México; 5 Rheumatology Department, Instituto Nacional de Rehabilitación “Luis Guillermo Ibarra Ibarra”, Mexico City, México

**Keywords:** Musculoskeletal ultrasound, Painful spastic hip, Children, Infantile cerebral palsy, Muscle thickness, Lower limb

## Abstract

**Background:**

In cerebral palsy (CP), spasticity is the dominant symptom and hip pain is one of the most common secondary conditions. Aetiology is not clear. Musculoskeletal ultrasound (MSUS) is a low-cost, non-invasive imaging technique that allows assessment of structural status, dynamic imaging, and quick contralateral comparison.

**Objective:**

A retrospective case-matched-control study. To investigate associated factors with painful spastic hip and to compare ultrasound findings (focusing on muscle thickness) in children with CP vs. typically developing (TD) peers.

**Setting:**

Paediatric Rehabilitation Hospital in Mexico City, from August to November 2018.

**Participants:**

21 children (13 male, 7 + 4.26 years) with CP, in Gross Motor Function Classification System (GMFCS) levels IV to V, with spastic hip diagnosis (cases) and 21 children age- and sex-matched (7 + 4.28 years) TD peers (controls).

**Characteristically data:**

Sociodemographic data, CP topography, degree of spasticity, mobility arch, contractures, Visual Analog Scale (VAS), GMFCS, measurements of the volumes of eight major muscles of the hip joint and MSUS findings of both hips.

**Results:**

All children with CP group reported chronic hip pain. Associated factors for hip pain (high VAS hip pain score) were degree of hip displacement (percentage of migration), Ashworth Level, GMFCS level V. No synovitis, bursitis or tendinopathy was found. Significant differences (p < 0.05) were found in muscle volumes in all hip muscles (right and left) except in the right and left adductor longus.

**Conclusion:**

Though possibly the most important issue with diminished muscle growth in CP children is the influence on their long-term function, it is likely that training routines that build muscle size may also increase muscle strength and improve function in this population. To improve the choice of treatments in this group and maintain muscle mass, longitudinal investigations of the natural history of muscular deficits in CP as well as the impact of intervention are needed.

## Introduction

Cerebral palsy (CP) is one of the most common physical and developmental disabilities, consisting of a heterogeneous group of permanent movement and posture disorders usually attributed to nonprogressive disturbances that occurred in the developing child’s brain. They are attributed to non-progressive brain lesions at different stages of development: prenatal, neonatal, and early post neonatal (before 5 years old)[1, 2].

The sustained contraction of anti-gravitational muscle groups and the accompanying weakness of their antagonists creates a biomechanical imbalance, which results in a longitudinal retraction of the muscle. For this reason, patients with spastic-type CP, by maintaining a sustained contraction of the adductor hamstrings, and flexor muscles of the hip, present a high risk of dislocation. Most children with severely displaced or dislocated hips experience pain [3–5]. The incidence of hip displacement in children with CP has been estimated at 35% while the risk of dislocation and pain increases with lower gross motor function (higher score of the Systematic Classification of Gross Motor Function, GMFCS)[6, 7].

The aetiology of pain sensations in the group with CP does not provide any clear indication why pain, associated with hip joint dislocation of spastic origin, occurs in some patients only. Pain is related to multiple factors and associated with functional limitations that make it difficult to sit, stand, or walk, and a lower quality of life in all domains [6, 8–13]. A systematic review showed that three out of four children with CP had high levels of pain, with severe limitations in mobility, interfering with activities of daily living, such as hygiene (changing diapers or bathing)[14].

Due to the depth of the hip joint and the limited number of examination manoeuvres, clinical evaluation of the hip is frequently limited. In children and the elderly, musculoskeletal ultrasound (MSUS) is used to diagnose, monitor, and treat musculoskeletal pathologies [15–17]. Because it does not require sedation, it is preferred to magnetic resonance imaging and permits a dynamic study of the structures as well as a rapid comparison of the contralateral structures without exposure to ionizing radiation [15, 16]. The anterior, lateral, and posterior parts of the hip can be seen with ultrasound. Particularly, periarticular soft tissue lesions, tendon pathology, osteophytes, synovial hypertrophy, synovial effusions, bursitis, and other bony irregularities can all be diagnosed [17]. Currently, pain improvement assessment strategies are carried out using qualitative instruments (hetero or self-applied scale), there is no quantitative measure of improvement in treatment in addition to pre-existing instruments; especially in patients who do not meet surgical criteria and have hip pain, MSUS is a useful tool, since it can detect tendinitis, bursitis, synovitis, peri trochanteric inflammatory changes or changes in muscles thickness that accompany hip pain and that can be managed with rehabilitation (i.e., botulinum toxin and / or chemo denervation) and in the future it can correlate changes in pain scores with the changes in the MSUS after treatment [18].

No previous studies were found that measured normal thickness in the hip muscles in children without pathology. The aim of the present study was to investigate the factors associated with hip pain in painful spastic hip (dislocated or subluxated) among children with CP and to compare the ultrasound findings with typically developing children.

## Materials and methods

A retrospective case-matched-control study was conducted at the Hospital de Pediatria del Centro Médico Nacional Siglo XXI (CMN S.XXI) in the period from August to November 2018, using a consecutive sampling method to form the two groups.

### Participants

Group 1 consisted of children with spastic CP. The inclusion criteria were: (a) verified CP diagnosis by a physician specializing in paediatric rehabilitation medicine, (b) with a spastic variant of cerebral palsy (bilateral spastic CP) (c) with levels 4 or 5 (GMFCS), (e) with a previous X-ray (for no longer than one week), where migration percentage was measured digitally in both hips by one of the authors (CGS) and (f) without previous treatment (chemo denervation or application of botulinum toxin injections to the lower limbs within the previous year). The exclusion criteria were: (a) children older than 15 years, (b) presence of hip deformity caused by trauma, infection, tumour, etc., (c) presence of neuromuscular disease other than CP; and 4) inadequately taken hip radiographs. Typically developing group consisted of children younger than 15 years without neurological- musculoskeletal disabilities and had no previous surgery to their lower limbs, who were recruited from the outpatient clinic of the same hospital and were included as a TD group. CP and TD groups were matched based on age and sex.

This study was performed in accordance with the principles of the Declaration of Helsinki. The study protocol was approved by Comité de Bioética del Hospital de Pediatría Dr Silvestre Frenk Freund Centro Médico Nacional Siglo XXI IMSS, IRB number: F/2018/3603/82). Informed consent was obtained from parents and / or guardians before inclusion in the study. In the same way, the researchers ensured that all minors, capable of understanding enough to participate in decision-making, gave their consent to participate in the research.

### Procedure

Both groups were recruited at the same time for both measurements (clinical and ultrasonographic). First, they went to their ultrasonographic assessment, and then, to their assessment with the rehabilitation physician (they were 3 physicians with 10 years of paediatric rehabilitation experience, each physician evaluates a child individually and aleatory) to carry out their clinical measurements. With a total time of one hour and 30 minuties for each patient in a period of August to November 2018.

We made clinical record sheet for the children with CP, that includes:

1. Age and gender.
2. Topography of the CP: Diparesis (affection of 4 extremities predominantly pelvic limbs),( and quadriparesis (affection of 4 extremities, including neck and trunk).
3. Degree of spasticity: Degree of resistance of as muscle to fast-passive movement. According to this scale, the first assessment is a slow-passive (›1.5 seg) movement (this manoeuvre helps us to identify a contracture in a specific joint), then the second assessment is a fast- passive (≤ 1 seg) movement (this manoeuvre helps us to identify spasticity, and is compared according to the modified Ashworth scale (MAS). Moderate to high inter and intra-rater reliabilities were reported in a recent meta-analysis, with higher reliability for upper than lower extremities. The scale is as follows: 0 (no increase in muscle tone), 1 (slight increase in muscle tone, manifested by minimal resistance at the end of the range of motion -flexion or extension-), 1+ (slight increase in muscle tone, manifested by a catch followed by minimal resistance throughout), 2 (marked increase in muscle tone, manifested by a catch in the middle range and resistance throughout the remaining range of motion, but affected part easily flexed, 3 (considerable increase in muscle tone, passive movement difficult) and 4 (rigid in flexion or extension)[19]. Degrees of spasticity in the following two muscle groups: hip adductors, hip flexors.
4. Mobility arch: analysed according to the range of motion (ROM) measured by goniometry. Patient in supine position, five measures were taken: hip flexion, hip abduction, adduction, internal rotation, and external rotation. And lateral position for de hip extension.
5. Degree of contracture (measured by goniometry). Patient in supine position, three measures were taken: hip flexors, hip adductors, and hamstrings: considering 0–30 degrees physiological and > 30 degrees inability to walk.
6. The intensity of pain at the moment of move de hip in their different range of motion, according to age and cognitive development, using the Visual Analog Scale (0–10) if the child can answer, where 0 is no pain and 10 is the most intense pain that you have felt in your life or, through the Wong-Baker FACES® Visual Analog Scale (VAS), composed of six drawn faces (identification according to the pain sensation represented graphically) with scores ranging from 0 to 10, in the case of the child do not have de capacity for answer, so the parents and physician take a score according at the face of the child at the time of move the hip. For the present study, we categorized hip pain scores 1 to 3 as mild, 4 to 7 as moderate, and 8 to 10 as severe hip pain.
7. Gross motor function measured using GMFCS, a 5-level classification system based on their self-initiated movement with particular emphasis on sitting, walking, and wheeled mobility: level IV children and youths are more likely to be transported in a manual wheelchair or use powered mobility and level V, individuals have severe limitations in head and trunk control and require extensive assisted technology and physical assistance [7].
8. The degree of hip displacement, measured with the percentage of migration in both hips using Reimers’ method, which varies from 0 to 100%, previously diagnosed by a hip x-ray. Percentage of femoral head coverage (defined as acetabular width divided by femoral head diameter, multiplied by 100) in relation to acetabulum was calculated using ultrasound [20].
9. The MSUS findings of both hips were carried out using an Esaote MyLab 25® Equipment, with a 7.5–12 MHz multi-frequency linear transducer, gain between 50 and 70%. Ultrasound examination was performed in two orthogonal planes: a longitudinal view in the standard plane at rest (patients were supine with the hip in neutral position) and a transverse view of the flexed hip with and without stress (if supine position was uncomfortable, slight flexion in the hip was obtained with a pillow behind the knees)[20]. The thickness of the muscle was measured, using calliper equipment, taking as reference the visible intermuscular fascia of each muscle studied, drawing a straight line towards the upper visible margin. Thickness measurements were taken in the muscular belly, of the iliopsoas, sartorius, rectus femoris, adductor magnus, adductor longus, adductor brevis, gluteus medius, and gluteus minimus muscles; bilaterally and in two orthogonal planes; using the best longitudinal image of each muscle as a standard measurement. Additionally, the hip joint was evaluated to rule out synovitis. To carry out a systematic study, the principles of the EULAR Guidelines for the hip joint and trochanteric region were followed, and two-plane evaluation for muscle evaluation [21, 22]. The live clips were exported and randomly numbered in a DICOM file. Three independent investigators took the images, and two independent investigators examined the images. One was an ultrasound expert with 18 years’ experience in musculoskeletal ultrasound (A; CHD) and the other a master´s student with 2 years, training in musculoskeletal ultrasound (B; AVMS). The performance of the two investigators was examined with interobserver variation.

The clinical record sheet for the children with TD, includes: age, gender, mobility arch, degree of contracture, normal hip x-ray, MSUS findings of both hips.

### Statistical analysis

Interobserver analysis was estimated using interclass correlation coefficients (ICC), with one-way random effect. The reliability is regarded as excellent if ICC 0.90, fair to good between 0.5 and 0.75, and poor if less than 0.5 [23].

Observer agreement. Interobserver agreement between ultrasound investigators showed a good to excellent correlation (Global ICC = 0.84, 95% Confidence interval = 0.663–0.955).

Shapiro–Wilks tests of normality found that the measured volume data was normally distributed for all muscles (p < 0.05) and required parametric statistics. Demographic and clinical data between CP and TD groups were compared with Pearson chi-square tests (for categorical outcome variables) and independent t-test, (for continuous outcome variables). Associated factors for hip pain (mild, moderate and severe) on CP group were compared with Pearson chi-square tests (for categorical outcome variables) and one way ANOVA, (for continuous outcome variables). Left and right hip muscle measurements on moderate and severe pain were compared with independent t-test.

All tests were 2-sided. A significance level of 0.05 was chosen. Statistical analysis was performed with IBM® SPSS® Statistics (version 26, IBM Corporation, New York, NY).

## Results

A total of 42 subjects (84 hips) were included. 61.9% were male (n = 26) and 38.1% female (n = 16), with a mean age of 7.02 (SD = 4.22) years (range 1–15 years). Case group consisted of 21 children with spastic CP (mean age: 7 + 4.26 years, 13 males, 8 females, while control group consisted of 21 children age- and sex-matched without neurological and musculoskeletal disabilities (average age: 7 + 4.28 years) (see Table 1). All children in case group reported chronic hip pain [mean Visual Analog Scale (VAS) 3.61 + 2.95 in the left hip and 2.61 + 2.41 in the right hip]. Pain was bilateral in 19 participants and unilateral in 2. Severe hip pain (score 7–10) was present in five patients, whereas seven had moderate hip pain (score 4–6) and nine had mild hip pain (score 1–3). MSUS measured a significant decrease in all hip muscles (right and left) except in the right and left adductor longus, highlighting the hypertrophy of the left adductor longus muscle in very young children (2–5 years) (Table 1). However, no tendinitis, bursitis or tendinopathy was found in any area evaluated bilaterally.

**Table 1:** Demographic and clinical characteristics of case and control groups

Demographic characteristics			
Parameter	Case (n = 21)	Controls (n = 21)	p value
	Mean (SD) n (%)		
**Sex** Boys Girls	13 (61.9) 8 (38.1)	13(61.9) 8 (38.1)	1.000^a^
**Age**	7(4.26)	7.04 (4.28)	.910^b^
**Clinical characteristics**			
**Hip pain (Visual Analog Scale (VAS)** **Mean (SD)**			
Left hip	3.61(2.95)	0	< 0.001^b^
Right hip	2.61(2.41)	0	< 0.001^b^
**Degree of hip displacement (percentage of migration)** **Mean (SD)**			
Left hip	44.09 (30.79)	0	< 0.001^b^
Right hip	41.86(29.72)	0	
**Hips classification according migration percentage, most displaced hip** **Mean (SD)**			
Normal < 33%	6 (28.6)	21	
Subluxated 33–89%	12 (57.1)	-	
Dislocated ≥ 90%	3 (14.3)	-	
**Degree of spasticity (MAS)** **Mean (SD)**			
Level 0	0	-	-
Level 1	0	-	-
Level 1+	4 (19.1)	-	-
Level 2	11 (52.3)	-	-
Level 3	5 (23.8)	-	-
Level 4	1 (4.76)	-	-
**Degree of contracture** **Mean (SD)**			
Left hip flexion contracture	21.38 (13.30)	0	< 0.001^b^
Left hamstring contracture	45.09 (22.25)	9.31 (6.03)	< 0.001^b^
Right hip flexion contracture	18.95 (10.84)	1.08 (5.21)	< 0.001^b^
Right hamstring contracture	44.85 (21.07)	11.5 (12.1)	< 0.001^b^
**Topography**	**Mean (SD)**		
Diparesis	17 (80.9)	-	-
Quadriparesis	4 (19.1)	-	-
**Gross Motor Function**	**Mean (SD)**		-
GMFCS level IV	15 (71.4)	-	-
GMFCS level V	6 (28.6)	-	-
**Left hip mobility arches** **Mean (SD)**			
Left flex	105.09 (25.9)	120	< 0.001^b^
Left abduction	23.95 (11.67)	45	< 0.001^b^
Left internal rotation	35 (19.3)	40	< 0.001^b^
Left external rotation	42.76 (24.03)	45	< 0.001^b^
Left adduction	25.09 (7.96)	30	< 0.001^b^
Extension left hip	5.28 (14.47)	30	< 0.001^b^
**Right hip mobility arches** **Mean (SD)**			
Right flex	104.6 (23.5)	120	< 0.001^b^
Right abduction	20.33 (10.05)	45	< 0.001^b^
Right internal rotation	39.50 (16.63)	40	< 0.001^b^
Right external rotation	41.45 (22.58)	45	< 0.001^b^
Right adduction	26 (6.42)	30	< 0.001^b^
Extension right hip	1 (16.53)	30	< 0.001^b^

(a) – Chi-square test. (b) – independent t-test

There was no significant difference between the prevalence of hip pain in quadriparesis vs. diparesis. Parameters significantly associated with hip pain, high VAS hip pain score, were: (1) degree of hip displacement (migration percentage) in both hips (right, p = 0.012 and left p = 0.001), (2) Ashworth Level (p = 0.021) (3) Degree of contracture (Left hip flexion contracture (p = 0.047) and (4) GMFCS level V (p < 0.001) (Table 2).

**Table 2:** Associated factors for hip pain (mild, moderate and severe) on CP group (n = 21)

Factors	Mild hip pain (n = 9)	Moderate hip pain (n = 7)	Severe hip pain (n = 5)	p value
	Degree of hip displacement (percentage of migration)			
Left hip. **Mean (SD)**	20.33 (16.07)	47.14 (22.58)	82.6 (17.85)	0.001^b^
Right hip. **Mean (SD)**	21.77 (14.18)	51.85 (24.36)	64 (37.81)	0.012^b^
	**Degree of spasticity (Ashworth Scale)**			
Level 0	0	0	0	0.021^a^
Level 1	0	0	0	
Level 1+	4	0	0	
Level 2	3	6	2	
Level 3	2	0	3	
Level 4	0	1	0	
**Degree of contracture** **Mean (SD)**				
Left hip flexion contracture	12.88 (11.12)	26.86 (14.32)	29 (6.55)	< 0.047^b^
Left hamstring contracture	36.66 (14.41)	43.28 (29.83)	62.8 (12.93)	< 0.51^b^
Right hip flexion contracture	14.89 (8.73)	18.85 (10.18)	27.6 (11.34)	< 0.173^b^
Right hamstring contracture	38.88 (14.49)	42.14 (27.64)	62.8 (12.93)	< 0.073^b^
**Topography**	**Mean (SD)**			
Diparesis	2	7	3	0.024^a^
Quadriparesis	7	0	2	
**Ambulation**				
GMFCS level IV	9	0	0	< 0.001^a^
GMFCS level V	0	7	5	
**Left and right hip muscle measurements**				
**Left Hip (milimeters)**				
**Mean (SD)**				
Sartorius	5.1 (3.31)	3.68 (1.87)	4.76 (1.91)	0.561 ^b^
Ischium	7.34 (4.37)	4.98 (1.96)	6.54 (2.72)	0.399 ^b^
Psoas	6.53 (4.12)	4.35 (2.07)	5.62 (2.61)	0.426 ^b^
Gluteus medius	3.02 (1.69)	2.99 (1.57)	3.94 (1.03)	0.505 ^b^
Gluteus minimus	3.57 (1.60)	3.24 (1.53)	3.92 (1.43)	0.761 ^b^
Long adductor	6.31 (3.15)	5.45 (2.74)	10.06 (4.25)	0.070 ^b^
Minor adductor	9.08 (4.86)	7.55 (3.19)	9.96 (2.01)	0.550 ^b^
Adductor magnus	6.07 (4.65)	5.76 (3.18)	10.16 (5.70)	0.212 ^b^
**Right Hip (milimeters)**				
**Mean (SD)**				
Sartorius	4.00 (1.57)	4.36 (2.29)	4.36 (1.85)	0.911 ^b^
Ischium	6.77 (3.69)	4.93 (2.30)	8.52 (3.26)	0.182 b
Psoas	5.99 (3.58)	4.16 (1.94)	6.14 (2.02)	0.369 ^b^
Gluteus medius	3.88 (1.88)	2.86 (1.41)	3.80 (0.31)	0.388 ^b^
Gluteus minimus	4.16 (1.82)	3.48 (1.55)	3.94 (0.76)	0.693 ^b^
Long adductor	7.08 (3.10)	7.48 (3.22)	9.92 (3.81)	0.307 ^b^
Minor adductor	8.62 (4.71)	6.67 (4.07)	8.64 (5.19)	0.667 ^b^
Adductor magnus	6.21 (4.63)	8.40 (4.18)	8.64 (5.11)	0.538 ^b^

(a) – Chi-square test. (b) ANOVA

Table 3 shows a significant increase in left long adductor and right ischium on MSUS measured between moderate and severe pain. No differences were found between mild vs. moderate and/or mild vs. severe (Table 2).

**Table 3:** Left and right hip muscle measurements on moderate and severe pain

	Moderate Pain n = 7	Severe Pain n = 5	p value
	Mean (SD)		
**Left Hip (milimeters)**			
Sartorius	3.68 (1.87)	4.76 (1.91)	0.177^a^
Ischium	4.98 (1.96)	6.54 (2.72)	0.139^a^
Psoas	4.35 (2.07)	5.62 (2.61)	0.184^a^
Gluteus medius	2.99 (1.57)	3.94 (1.03)	0.134^a^
Gluteus minimus	3.24 (1.53)	3.92 (1.43)	0.231^a^
Long adductor	5.45 (2.74)	10.06 (4.25)	**0.022** ^**a**^
Minor adductor	7.55 (3.19)	9.96 (2.01)	0.07^a^
Adductor magnus	5.76 (3.18)	10.16 (5.70)	**0.050** ^**a**^
**Right Hip (milimeters)** **Mean (SD)**			
Sartorius	4.36 (2.29)	4.36 (1.85)	0.498 ^a^
Ischium	4.93 (2.30)	8.52 (3.26)	**0.024** ^**a**^
Psoas	4.16 (1.94)	6.14 (2.02)	**0.050** ^**a**^
Gluteus medius	2.86 (1.41)	3.80 (0.31)	0.091^a^
Gluteus minimus	3.48 (1.55)	3.94 (0.76)	0.280^a^
Long adductor	7.48 (3.22)	9.92 (3.81)	0.129^a^
Minor adductor	6.67 (4.07)	8.64 (5.19)	0.239^a^
Adductor magnus	8.40 (4.18)	8.64 (5.11)	0.466^a^

a) independent t-test

**Fig. 1:**
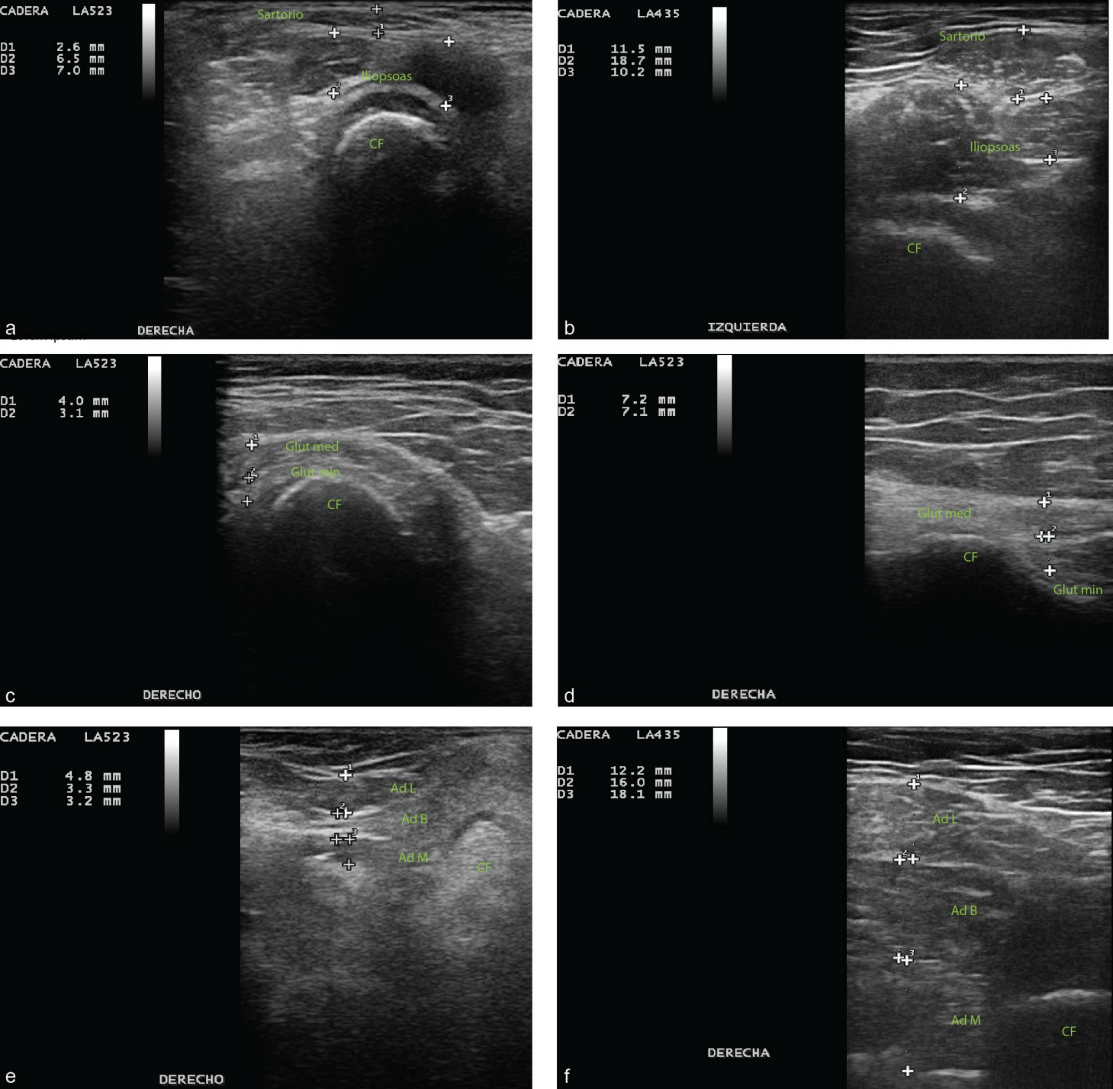
MSUS differences in thickness of sartorius, iliopsoas, gluteus medius, gluteus minimus, adductor longus, brevis and magnus muscles in a patient with a high degree of spasticity versus healthy control (**a**) sartorius and iliopsoas muscles in a patient with a high degree of spasticity. (**b**) sartorius and iliopsoas muscles in healthy control. (**c**) gluteus medius and minor muscles in a patient with a high degree of spasticity. (**d**) gluteus medius and minor muscles in healthy control. (**e**) adductor muscles longus, brevis and magnus in a patient with a high degree of spasticity. (**f**) adductor longus, brevis and magnus muscles in healthy control. CF = femoral head; Glute med = gluteus medius; Min glute = gluteus minimus; Ad L = Adductor longus; Ad B = Adductor Brevis; Ad M = Adductor magnus

Figure 1 shows MSUS differences in the thickness of sartorius, iliopsoas, gluteus medius, gluteus minor, adductor longus, brevis and magnus muscles on a patient with a high degree of spasticity versus TD.

Additionally, all images were evaluated to search for cortical and cartilage femoral head differences or abnormalities; however, it was not valuable to report since the femoral age at that age shows different degrees of maturity and cartilage thickness, and it was not the objective of this study.

## Discussion

Individuals with CP are severely physically impaired by significant muscular atrophy and in most cases hip pain and the aetiologies are poorly understood, yet some factors are more to be related [1, 24–29]. This study found an association between hip pain and:

1) Degree of hip displacement (percentage of migration): At a mean age of seven years, the average of children with CP have subluxated hips, increasing the risk for luxation. This is likely to occur, in children that are unable to walk. If hip displacement progresses to dislocation, the child may develop early onset arthritis and chronic pain.

Otherwise, we had children with CP with normal percentage of migration and mild hip pain; and other group of patients, with a similar migration percentage, they show moderate-severe pain. In those cases, we wonder what was the reason of pain, an what structures were implicated.

2) Degree of function (GMF V): as functional deterioration and physically impaired are more severe, perception of pain and risk of luxation increases 70–80%. Similar results have been shown from the Victorian Cerebral Palsy register,

3) Restriction in all hip range of motion in patients with CP, compared with typically developed children, cause musculoskeletal disorders, contractures and chronic pain.

4) Spasticity in the muscles around the hip joint, and 5) patients with moderate vs. severe hip pain had significant increase in left long adductor and right ischium on MSUS measured, which correlates with muscles that intervene on the adduction of de hip.

Pain in children with CP has wide-ranging consequences, so early recognition is essential. This significantly reduces quality of life, affects mental health, impaired sleep and causes less participation in activities of daily living (e.g. relationships and recreation) [32, 33].

In childhood pain is a predictor of restricted participation. Motor disorders of CP are often associated with musculoskeletal abnormalities, hip displacement is the second most common. Hip pain is a frequent phenomenon with a reported prevalence of 27 to 77% in patients with CP, this variation depends on the research criteria, time period, age, CP type and GMF level [11, 34, 35]. Some authors report that it is related by itself to migration or dislocation (as that found in some patients in the present study), however some patients have a migration percentage lower than 33% and still report pain [33–35]. Additionally, there were no discernible variations in the percentage of migration between individuals who reported moderate pain and those who reported severe pain. Other studies consider osteoarthritis and femoral head deformity as an additional factor. On the other hand, we found a correlation between the degree of spasticity (Ashworth Scale Level) and pain, contrary to that reported in the literature, although the information is not conclusive [19, 34].

Hip displacement can lead to pain, reduced function, and quality of life; this displacement is due to spasticity and contraction of the hip adductors and flexors, as well as the medial hamstrings, with atrophy of the hip extensor and abductor muscles, resulting in muscle imbalance and bony deformity (increased femoral anteversion and acetabular dysplasia).

Hip muscle thickness has not been clarified on individuals with CP vs. TD. Having measurements of muscle thickness is valuable because it may, in the future, allow doctors to rule out other causes of pain, that are not previously considered in the literature. In our study, no data of joint inflammation or soft tissue injuries were found.

In this study we assessed the thickness of eight hip muscles and we find out that all of them were significantly smaller in the group of children with CP than in the group of TD children of comparable age and sex, except the right and left adductor longus. We found studies with measurements of some lower extremity muscles and their relationship with gait, but not with pain [30, 31, 36, 37].

Some studies report changes in muscle thickness associated with spasticity and stiffness, that may be due to the musculoskeletal alterations in CP [14, 24, 28]. Due to the important relationship between muscle thickness and functional capacity (GMF), in individuals with CP, recent studies have focused on the evaluation of both: patient function and muscles and tendons characteristics [14, 24, 28]. These studies have reported critical changes in the muscles that cannot be justified by neurological changes alone [37]. Consequently, a better understanding of the alterations in the spastic muscles is of particular importance, especially for the search and treatment of its cause.

The force-generating capacity of a muscle is mainly due to its morphological and architectural characteristics [36, 38]. The thickness of the muscle was valued as an approximation of the size of the muscle, due to its high correlation with the cross-sectional area of ​​the same muscle, highlighting the reduction in thickness in the hip muscles except for the long adductors [39–41]. Therefore, we believe that these alterations could be an inadequate adaptation of the muscle architecture, trying to maintain function as much as possible, having a negative influence on joint control, and facilitating the loss of muscle strength. This study highlights the role of the MSUS to evaluate the hip, despite the lack of previous studies using this imaging method to evaluate the muscle dynamically and in real time among patients with ICP.

One of the weakness of the study was the assessment of pain, which was based on self-report with a numerical or visual analogue scale (pain is understood as a participatory experience), using self-report as the standard criterion [42]. We recruit a representative group of children with CP who have not had significant intervention (surgical intervention, botulinum toxin injections, etc.) during their lifetime. However, due to some of the comorbidities and/or ages of the patients, self-report was not always possible. By contrast, the indirect reports reported in other studies did not assess pain in this way, therefore, given the subjective nature of the pain, the assessment was not accurate and could have overestimated or underestimated pain [12, 43]. No studies were found evaluating changes in muscle thickness in affected patients associated with pain. The use of the MSUS, performing a dynamic examination, with a relatively low cost, wide availability, without the need for sedation and lack of ionizing radiation [21, 22] facilitated the evaluation of the patient in a pain-free environment.

This study is cross –sectional, so it cannot infer causality. On the other hand, the vision of the ultra-sonographer is modificated because of the operator-dependent bias, but we can still find the muscle structures despite the patient spasticity and difficulties to access and measure some studied areas.

The clinical implications of the study, related to the modification of muscle thickness in patients with CP and painful spastic hip, are the initial step to find the causes of these changes and early detection of them through the MSUS; and even to determine if these changes occur before or after hip subluxation or dislocation.

Currently, the effectiveness of the different treatments in painful spastic hips are carried out using qualitative self-applied or hetero-applied instruments, and there is no quantitative measurement of pain improvement. It is important to carry out prospective, randomized, controlled, double-blind studies with methods of imaging with additional technology such as elastography, muscle mapping with other techniques; and evaluation of treatment, by assessing it effects at the structural and functional level. This study opens new opportunities to improve the early diagnosis of musculoskeletal injuries, the quality of life of children with CP and spastic hip, start early treatment, and improve prognosis in the medium and long term. To choose properly treatments in this group and maintain muscle mass, longitudinal investigations of the natural history of muscular deficits in CP as well as the impact of intervention are needed.

## Conclusions

Though possibly, the diminished muscle growth in PC children, influence their long-term function. It is likely that training routines, building up muscle size, increasing muscle strength will improve function in this population. The low muscle reserves could expose individuals to harmful consequences of muscle aging.

It’s a common complain that pain in CP people is underdiagnosed and undertreated; the approach of pain is complex and a challenge, due to the existence of many pain causes and difficulties in communication. In this study, the causes of pain, were related to the degree of hip dislocation, spasticity, and structural changes by MSUS, which showed a direct association with structural muscle architecture (decrease in its thickness). More studies are required to identify risk groups, standardize hip muscle imaging and measurement techniques, to enhance an early treatment and avoid or delay quality of life deterioration.

In order to prevent painful musculoskeletal complications of CP, in patients with spastic hips, prior to subluxation, periodic muscle evaluations should be done. It is possible that the MSUS can provide surveillance of musculoskeletal findings to prevent complications in the medium and long term.

Hip surveillance is defined as the process of monitoring and identifying early critical indicators of hip displacement. In addition to identification, early intervention in these types of hips can reduce the number of reconstructive surgeries, and reduce negative effects on their daily activities, participation, quality of life, and mental health.

## Data Availability

The datasets used and/or analysed during the current study available from the corresponding author on reasonable request.
